# Innovation and economic crisis in transition economies

**DOI:** 10.1007/s40821-021-00192-y

**Published:** 2021-11-10

**Authors:** Katharina Friz, Jutta Günther

**Affiliations:** University of Bremen, Faculty of Business Studies and Economics, Max-von-Laue-Str. 1, 28359 Bremen, Germany

**Keywords:** Innovation behavior, Economic downturn, Transition countries, O12, O14, O30, O31, O57

## Abstract

Based on Schumpeterian theoretical considerations, this paper investigates the innovation behavior of firms during the severe economic crisis of the year 2008/2009. It focuses on transition countries of Central and Eastern Europe and Central Asia, which have completely restructured their innovation systems through the course of transition from planned to market economies a relatively short time ago. As a result of the crisis, we observe a strong decline of innovation activity in all transition economies. In line with the literature, there is, however, empirical evidence for both creative destruction as well as creative accumulation. This underlines two key findings: firstly, the universality and durability of Schumpeterian assumptions, and secondly, a call for anti-cyclical innovation policy.

## Introduction

**Fig. 1:**
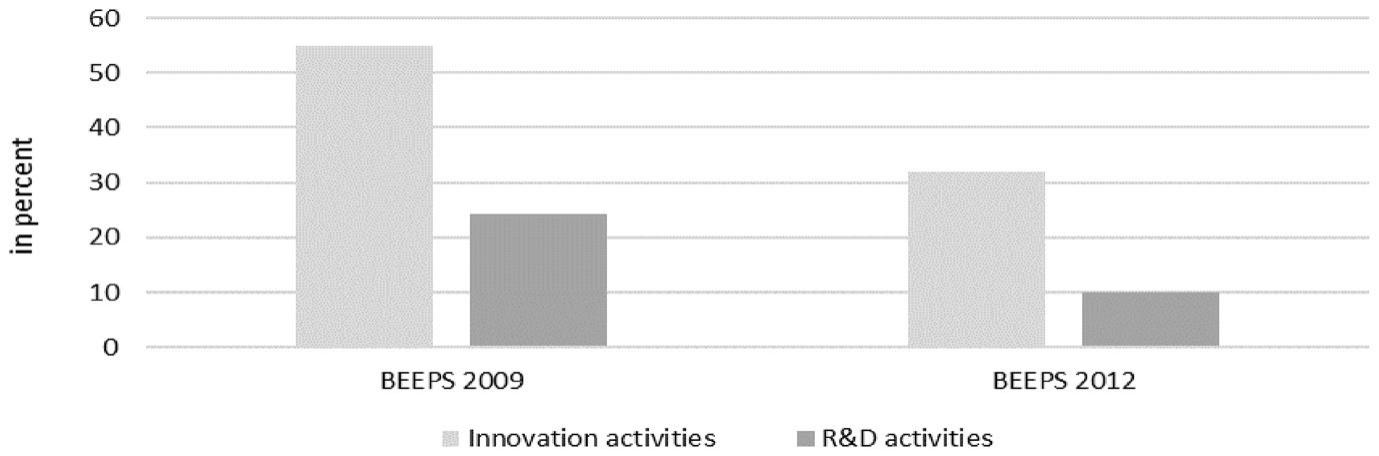
Share of firms (in % of all firms) involved in innovation or research and development (R&D) activities. Note: Data includes 29 transition economies. The BEEP survey 2009 and 2012 refer in each case to innovation/R&D activities in the period 2005–2007 and 2009–2011, respectively. Source: European Bank for Reconstruction and Development

The global financial crisis (GFC) of 2008/2009 had catastrophic repercussions on individual countries as well as on the international economy (Crotty, 2009; Obstfeld & Rogoff, 2009). Like many developed and emerging economies, Central and Eastern Europe (CEE) were hit hard by the GFC (Fagerberg & Srholec, 2016). Using firm level data of the Business Environment and Enterprise Performance Surveys (BEEPS), we can see that the economic crisis in CEE was also accompanied by a strong reduction of research and innovation activities. Considering 29 economies in CEE and the Commonwealth of Independent States (CIS) and comparing 2005–2007 and 2009–2011, we observe a significant drop in research and innovation activities (see Fig. 1).

Descriptive statistics give a first impression about innovation and research activities before and after the economic crisis. Overall, it seems that innovation appears more cyclical rather than anti-cyclical in these countries. However, the reaction of individual firms may be different depending on their economic and financial situation as well as business strategy and other firm specific circumstances. Therefore, this paper will empirically investigate the determinants of firms’ innovation and research activities in times of a deep economic crisis. In this context, we also scrutinize how the GFC of 2008/2009 shifted the innovation behavior of companies in the sense of creative destruction or accumulation. The subject of the paper is of great importance, given the fact that the world economy is again experiencing an economic crisis triggered by the COVID-19 pandemic. Continuous innovation efforts are not only crucial for a company’s long-term economic performance but also for a country’s knowledge base and its long-term growth (Grossman & Helpman, 2001; Romer, 1986, 1990). Schumpeterian literature suggest that innovative activities and innovative organizations can be revamped by economic crises through the effects of creative accumulation and destruction (Schumpeter, 1934, 1939). Moreover, creative destruction can be an opportunity for more economic diversification that benefits social welfare. Previous evidence indicates that diversification is particularly important for social welfare in transition economies (Ali & Cantner, 2020).

Whilst there exist several studies for European Union and Latin American economies on innovation behavior during the GFC, no empirical insights are available for transition economies so far. We use the term “transition economies”, referring to the formerly planned economies in CEE and the CIS. We acknowledge that transition in the sense of institutional change from a planned to a market economy has formally been completed in many of these countries, and that the group of all 29 transition economies today is quite heterogeneous. Conversely, these countries share the common experience of system break and complete restructuring of their economic and technological system. Moreover, these relatively young market-based innovation systems may be more vulnerable compared to established market economies. It is therefore very likely that the GFC of 2008/2009 had a stronger impact on innovation activity in these countries. Harmonized company survey allows a comprehensive analysis in which heterogeneity will be taken into consideration. Our paper is not focusing on a specific industry sector or technology, but how an economic crisis affects firms’ innovation performance in general.

According to our findings, the crisis leads to an overall decline in innovation activities. Moreover, a shift of innovation activities from small to large firms occurs which indicates creative accumulation. However, we also observe that young firms increase their likelihood to innovate after the crisis which gives some weak indication for creative destruction as well. Further, our results show firms engaged in R&D activities innovate more persistent and thus are less affected by the GFC. Additionally, firms with access to financial resources such as loans and subsidies have a higher likelihood to innovate after the crisis.

The paper is organized as follows: Sect. 2 provides the literature review as well as the hypotheses. Section 3 describes the data samples and the econometric model. The empirical results are presented in Sect. 4. Finally, Sect. 5 provides a brief summary, dealing with study’s limitations and a conclusion.

## Theoretical considerations, literature review and hypotheses

In the 2008/2009 crisis, innovation activities overall declined significantly because of low demand expectations and increased uncertainty (Archibugi et al., 2013a, b; Kanerva & Hollanders, 2009; OECD, 2009, 2012). Schumpeter argued that an economic turmoil could provide the chance for firms to become more efficient and innovative through creative destruction, allowing them to even gain competitive advantage (Schumpeter, 1911, 1934). Creative destruction is characterized by low learning cumulativeness, high technological opportunities and a dynamic environment with higher entry and exist rates (Archibugi et al., 2013a; Francois & Lloyd-Ellis, 2003; Malerba & Orsenigo, 1995). These more agile and flexible structures within new entrants and small companies allow them to better adapt to an economic downturn, challenging incumbent firms. Incumbent firms, though, perform research and development (R&D) and innovation as routine activities because they build on their previous knowledge in specific (technological) areas (Schumpeter, 1942). This innovation process is called creative accumulation and is characterized by path-dependent patterns, high knowledge accumulation, low opportunities and high entry barriers which lead to a more stable environment (Archibugi, 2017; Archibugi et al., 2013a; Breschi et al., 2000; Nelson & Winter, 1982; Schumpeter, 1942). Hence, established companies benefit from their path-dependent patterns and cumulative learning processes and innovate continuously unaffected by economic fluctuations.

The most recent literature investigated firms’ innovation behavior during the GFC 2008/2009 (Antonioli & Montresor, 2021; Archibugi et al., 2013a, b; Filippetti & Archibugi, 2011; Paunov, 2012). Archibugi et al. (2013a) examining panel data from 2500 British firms, found that firms classified as great innovator are more likely to increase innovation during the crisis (but not before) and thus supporting the case of creative accumulation. They also find evidence that new fast-growing firms are as well more likely to expand their innovation investment, indicating a process of creative destruction. However, the empirical evidence is not yet conclusive. Archibugi et al. (b) analyzing survey data of 5238 European companies from 2009, find that small or new firms are more likely to increase their investment in innovation during the GFC, while before the crisis larger firms are more likely to increase their investment in innovation. Thus, they conclude that even though before the crisis creative accumulation prevailed, during the recession firms’ innovation behavior converge towards creative destruction. Findings from other studies, however, display the opposite. Teplykh (2018) using panel data from 420 Western European firms, found that larger firms innovated more during the crisis, while small firms struggled the most, indicating a stronger tendency toward creative accumulation. This is in line with Correa and Iootty (2010) who show for 1686 Eastern European firms that young and innovative firms are more affected by GFC. Paunov (2012) confirms this for 1548 Latin American firms, which are also an example of how young firms are less likely to innovate in times of crisis. In these studies, liquidity constraints are a listed reason for the innovation weakness of small firms during an economic slump because smaller or younger companies have more difficulties to access external finance due to small credit history (Correa & Iootty, 2010; Paunov, 2012; Teplykh, 2018). In fact, getting access to external finance during an economic downswing becomes difficult for firms because banks, markets and investors are more risk averse in recessions (OECD, 2009, 2012; Paunov, 2012). These financial constraints detain innovation during recessions (Aghion et al., 2012; Hyytinen & Toivanen, 2005; Stiglitz, 1993).

The most recent empirical literature based on studies of European and Latin American countries indicates that there is no pure cyclical or anticyclical innovation behavior (Archibugi et al., 2013a, b; Filippetti & Archibugi, 2011; Paunov, 2012). It further demonstrates that creative destruction and creative accumulation co-exists. However, it should be noted that the countries studied so far are at different stages of development, which could explain the discrepancies in the results. In transition countries, not much is yet known about the impact of the GFC on innovation behavior. All transition economies experienced a system break with heavy losses of their scientific and industrial research and development (Meske, 2000). Since the 1990s, they have tried to build-up and modernize their innovation systems and to re-engage in original technological activities (Dyker, 2010; Günther, 2015; Varblane et al., 2007). The economic crisis 2008/2009 puts these achievements at risk. Using firm-level data for a large number of transition economies and drawing on Schumpeterian theoretical considerations, we will test the following hypotheses about firm behavior in transition economies for the pre- and post-crisis periods.

According to the literature, incumbent firms in general profit from their established resources and are more robust in innovating during an economic crisis (Archibugi et al., 2013a; Paunov, 2012; Teplykh, 2018). In transition economies, it must also be accounted for that the institutional environment often fosters the success of large firms while the opportunities for small and medium companies are restricted (Golikova & Kuznetsov, 2017). Furthermore, incumbent firms are former organizations of the planned economy. They survived by adapting to market conditions and a changing institutional environment which completely disrupted their innovation routines (Maksimov et al., 2017; Radosevic & Auriol, 1999). This profound experience may have given these companies a greater resilience to other crises. Therefore, the first hypothesis is:

**H1**: The crisis leads to a shift of innovation activities across firms towards larger or older firms (in the sense of creative accumulation).

However, a crisis can provide chances for small and new firms to emerge and gain market power through creative destruction (Archibugi et al., 2013a; Francois & Lloyd-Ellis, 2003; Malerba & Orsenigo, 1995). In transition economies, these young firms have no predecessor in the pre-reform economy and emerged in an already competitive environment, which is expected to make them more responsive to changing market conditions (Carlin et al., 2004). Thus, the second hypothesis is formulated as follows:

**H2**: The crisis leads to a shift of innovation activities across firms towards small or younger firms (in the sense of creative destruction).

Financial constraints are one of the main reasons to cut back innovation during an economic downturn (Hyytinen & Toivanen, 2005; Spatareanu et al., 2019; Stiglitz, 1993). The results of Gorodnichenko and Schnitzer (2013) and Mateut (2018) show that this also applies for transition countries in Eastern Europe and Central Asia. Furthermore, during a crisis banks, markets and investors become more risk averse and it is more difficult to get access to external finance (OECD, 2009, 2012; Paunov, 2012). Hence, the third hypothesis to be tested is:

**H3**: Firms with better access to finance are less likely to cut back their innovation activities during the crisis.

**H3**a: Companies with better access to finance are more likely to spend money on R&D and are therefore more likely to innovate during the crisis.

## Data and econometric specification

### Description of the data

The analysis makes use of the Business Environment and Enterprise Performance Survey (BEEPS) which is implemented by the EBRD (European Bank for Reconstruction and Development) in partnership with the World Bank. The BEEPS data is a firm-level survey based on face-to-face interviews with managers containing information on a wide range of standard firm characteristics. BEEPS also covers a wide range of business environment topics. Furthermore, it provides the advantage that firms self-report various types of their innovation activity such as: if the company introduced new products or services or did a major upgrade of existing ones or acquired a new production technology over the last 3 years. ‚New’ in this case means new to the firm, not necessarily new to the market. A frequently used alternative in innovation research is a combination of firm and patent data. We have not taken this approach because analyzing patent activity in transition countries is less suitable since firms are more likely to innovate through imitation or adaptation instead of inventing completely new (patentable) things of the existing state-of-the-art technologies (Acemoglu et al., 2006; Aghion et al., 2002; Gorodnichenko & Schnitzer, 2013; Gorodnichenko et al., 2009). Using publication data is another alternative. However, this leads to the problem of language bias, as publication databases typically only include English-language publications.

We analyze the fourth and fifth wave of the BEEPS that were conducted in 30 countries^1^ during 2009 and 32 countries^2^ during 2012. The surveys contain answers from almost 12,000 enterprises in 2009 and 15,600 in 2012. Since our research concentrates only on transition countries we have omitted data from Turkey, Greece and Cyprus. Our final sample comprises 10,846 observations in 2009 and 14,539 in 2012 for 29 transition countries. Both surveys have a similar sampling frame and contain a wide range of identical questions. Each sample includes very small firms with a minimum of two employees as well as large firms with up to 10,000 employees. The sample excludes companies that are ruled by government price regulations such as electric power, gas and water supply and companies that are 100% state-owned. Overall, the sample frames have been designed by a stratified random sampling to assure a representative structure of the firms’ population in each country. In each country, the sectoral composition concerning the share of manufacturing firms versus firms in services has been set by their contribution to country’s GDP.^3^ Furthermore, the data includes companies from both rural areas and large cities. Moreover, each questionnaire includes a question regarding the firms’ innovation activities over the last 3 years.^4^ This enables us to compare innovation behavior before and during the aftermath of the crisis.

We rely on pooled data for data-related reasons. Due to missing information about panel firm identification numbers, a unique firm identification in both waves is not possible. Moreover, small panel data set of heterogeneous firms makes it difficult to determine robust relationships (Gorodnichenko & Schnitzer, 2013).

### Operationalization of key variables

To investigate our first two hypotheses, we use the following firm characteristics: firm size measures the number of full-time employees (at the end of the fiscal year) and ranges from micro, small, middle to large firms. The size categories are in accordance with the OECD’s criteria.^5^ Further, age is measured as the number of years since the firm is operating and coded as a categorical variable [from 1 = start-up (1–5 years) to 4 = incumbent (over 21 years)]. We included the sub-categories start-up to control for newly created businesses as the first 5 years are the most challenging years for a company (Fort et al., 2013). Alternatively to firm age, we include categories of manager experience measured in years. With respect to the third hypothesis, the firm’s financial situation is described through the dummy loan (if the firm has currently a loan from a financial institution or not). In addition, we include a subsidies dummy (if the firm received governmental subsidies over the last 3 years or not), as subsidies can help stimulating firm’s innovation activities in times of crisis (Brautzsch et al., 2015; Mateut, 2018; Paunov, 2012). As a measure of firm financial constraints, we use the following two variables: (1) the dummy variable overdue, which indicates if the firm has overdue payments by more than 90 days or not. (2) Self-reported problematic to get access to finance, which includes availability and cost, interest rates, fees and collateral requirements. Access to finance is coded ‘1’ if it is none to minor obstacle, ‘2’ if it is a moderate obstacle and ‘3’ if it is a very severe to major obstacle.

Furthermore, we include R&D as a measure of innovation input, even though not all R&D activities generate innovations necessarily. The dummy R&D (inhouse or outsourced) measures whether a company spends money on R&D or not.^6^ The variable employee growth is included as firms’ employment decisions can reflect the effects of an economic plunge. Moreover, a firm is foreign owned if the foreign shareholder holds more than 50%. Gorodnichenko and Schnitzer (2013) and Karymshakova et al. (2019) found that foreign-owned companies innovate more in transition countries than local firms. As foreign competition and exporting status can have an impact on firm behavior (Beneito et al., 2015; Gorodnichenko et al., 2009; Mateut, 2018; Molodchik et al., 2021) we include export defined as 1/0 if the company is doing export business. Background measures the firms’ origin: 1 = private from the start, 2 = privatized, and 3 = other (e.g. private subsidiary of a formerly state-owned firm, joint venture with foreign partner). The ordinal variable education describes the share of employees with a university degree and captures the human capital within a firm.

### Summary statistics

Table 1 reports the summary statistics of all variables for each survey wave. Among the central explanatory variables, the share of firms classified as micro and small increase in Beeps 2012 compared to 2009, while the share of medium and large firms slightly declines. A possible explanation is that firms were forced to dismiss employees due to the GFC. The share of firms involved in R&D sinks by almost 15 percentage points in BEEPS 2012 compared to 2009. Other financial indicators also decrease in the 2012 survey, as expected. The percentage of firms with a current credit line drops by about 10 percentage points. It is surprising that the share of firms ranking access to finance as great obstacle decreases from BEEPS 2009 to 2012. The share of firms with overdue payments stays stable in both waves.

**Table 1:** Summary statistics

Variable	N	Mean	Std. dev.	Min	Max
**BEEPS survey 2009**					
Dependent variable					
Product or process innovation	10,828	0.637	0.481	0	1
Central explanatory variables					
Firm size	10,729	2.24	0.902	1	4
Firm age	10,839	2.671	0.864	1	4
Manager experience	10,839	2.775	0.987	1	4
R&D activities	10,717	0.243	0.429	0	1
General economic and financial situation					
Current loan	10,703	0.464	0.499	0	1
Subsidies	10,678	0.086	0.281	0	1
Employee growth	10,079	1.68	0.829	1	3
Sales growth	7321	1.403	0.574	1	3
Financial constraints					
Overdue payment	10,731	0.642	0.48	0	1
Access finance	10,396	1.817	0.849	1	3
Control variables					
Foreign owned	10,839	0.073	0.26	0	1
Background	10,823	1.347	0.598	1	3
Human capital (HC)	10,336	2.388	0.855	1	4
Export	10,806	0.233	0.423	0	1
EU transition	10,839	.316	0.465	0	1
**BEEPS survey 2012**					
Dependent variable					
Product or process innovation	14,536	0.433	0.495	0	1
Central explanatory variables					
Firm size	14,449	1.937	0.809	1	4
Firm age	14,537	2.656	0.912	1	4
Manager experience	14,538	2.792	0.955	1	4
R&D activities	14,443	0.102	0.302	0	1
General economic and financial situation					
Current loan	14,331	0.34	0.474	0	1
Subsidies	14,409	0.081	0.273	0	1
Employee growth	13,519	1.78	0.794	1	3
Sales growth	8555	1.518	0.5	1	2
Financial constraints					
Overdue payment	14,538	0.633	0.482	0	1
Access finance	14,250	1.567	0.793	1	3
Control variables					
Foreign owned	14,538	0.046	0.21	0	1
Background	14,523	1.207	0.514	1	3
Human capital (HC)	13,858	2.68	0.96	1	4
Export	14,403	0.191	0.393	0	1
EU transition	14,538	0.256	0.436	0	1

Source: European Bank for Reconstruction and Development

Table 5 presents the correlation coefficients. The coefficient suggests that larger firms are more engaged in R&D. Similarly, there is a positive correlation between firms involved in R&D and receiving subsidies. Human capital (HC) measured in form of employees with a university degree and R&D spending are only weakly positive correlated. There is no indication of multicollinearity problems.

### Econometric specification

The dependent variable in our analysis is binary and stands for product or service innovation or process innovation, with an either “yes, innovated over the last 3 years” or “no, did not” option. Innovation in this context is defined as the introduction of new products/services or process technologies.^7^ The query of firms’ innovation activities is in accordance with the Oslo Manual established by OECD and Eurostat. Due to the binary dependent variable, a logit model is employed for the estimation. We have chosen the logit approach as it facilitates the interpretation of the coefficients (Archibugi et al., 2013b). The vector of explanatory and control variables encompasses firm characteristics such as size, age, employee growth over the last 3 years, manager experience, R&D, education, subsidies over the last 3 years, and foreign owned.

We are aware of a possible reverse causality that has to be considered. Therefore, the estimated correlation between the various firm characteristics and innovation activities cannot be considered causal. Nevertheless, this paper attempts to determine as best as possible how size, age, R&D activities, and financial measures affect innovation through the variety of controls. To control for unobserved heterogeneity across countries and industry sectors we include country as well as industry dummies based on four-digit industry codes according to ISIC Revision 3.1 classification. An overview of the industry labels is provided in Table 6.

## Empirical results

### Baseline results

The main findings of the study are presented in Table 2. Column 1 shows the main variables of interest (firm age, firm size and financial measures) whilst in column 2, the age variable is expressed as the manager experience. All specifications control for industry and country fixed effects and cluster standard errors at industry and year level. In addition, a likelihood ratio test was applied to ensure that the models explain more than an empty base model. Models with a significant p-value (less than 0.01) are included.

**Table 2:** Logit estimation results of pooled BEEPS waves

	(1)	(2)
Size: small firm	1.152***	1.165***
	(0.0471)	(0.0482)
Size: medium firm	1.214***	1.242***
	(0.0667)	(0.0675)
Size: large firm	1.265**	1.317***
	(0.0956)	(0.103)
Age: young firm (6–10 years)	1.162**	
	(0.0591)	
Age: middle aged (11–20 years)	1.296***	
	(0.0579)	
Age: incumbent (> 20 years)	1.341***	
	(0.0885)	
Manager exp. (6–10 years)		1.079
		(0.0610)
Manager exp. (11–20 years)		1.320***
		(0.0740)
Manager exp. (> 20 years)		1.271***
		(0.0889)
RD activities	5.266***	5.273***
	(0.314)	(0.311)
Subsidies	1.351***	1.362***
	(0.0750)	(0.0760)
Current loan	1.289***	1.290***
	(0.0431)	(0.0426)
Overdue	1.250***	1.252***
	(0.0493)	(0.0494)
Employee growth increased	1.292***	1.265***
	(0.0546)	(0.0558)
Employee growth decreased	1.002	1.006
	(0.0372)	(0.0363)
Access finance: no/minor obstacle	0.984	0.983
	(0.0430)	(0.0427)
Access finance: great obstacle	1.257***	1.253***
	(0.0523)	(0.0519)
Foreign owned	1.301***	1.293***
	(0.0823)	(0.0811)
Export	1.392***	1.389***
	(0.0975)	(0.0981)
HC: up to 25% have university degree	1.199**	1.203**
	(0.0884)	(0.0873)
HC: 25–50% have university degree	1.354***	1.361***
	(0.102)	(0.101)
HC: more than 50% have university degree	1.531***	1.543***
	(0.113)	(0.113)
Private from start	1.060	1.036
	(0.0740)	(0.0695)
Privatization	0.840**	0.847**
	(0.0699)	(0.0683)
N	21,395	21,395
PseudoR	0.178	0.179
Log likelihood	− 12,030.574	− 12,023.846
LR Chi^2^	5221.32	5234.77
Prob > Chi^2^	0.000	0.000

The dependent variable is binary standing for process or product/service innovation activities. Reference groups: for firm size: micro firms; manager experience/age: 1–5 years; employee growth: unchanged; access finance: moderate obstacle; HC: no workers with university degrees. Time controls as well as country and industry fixed-effects are included. Exponentiated coefficients: to better interpret our results, we transform the coefficients into odds ratio; standard errors in parentheses are clustered at sector × wave level, *p < 0.10, **p < 0.05, ***p < 0.001; Source: European Bank for Reconstruction and Development

**Fig. 2:**
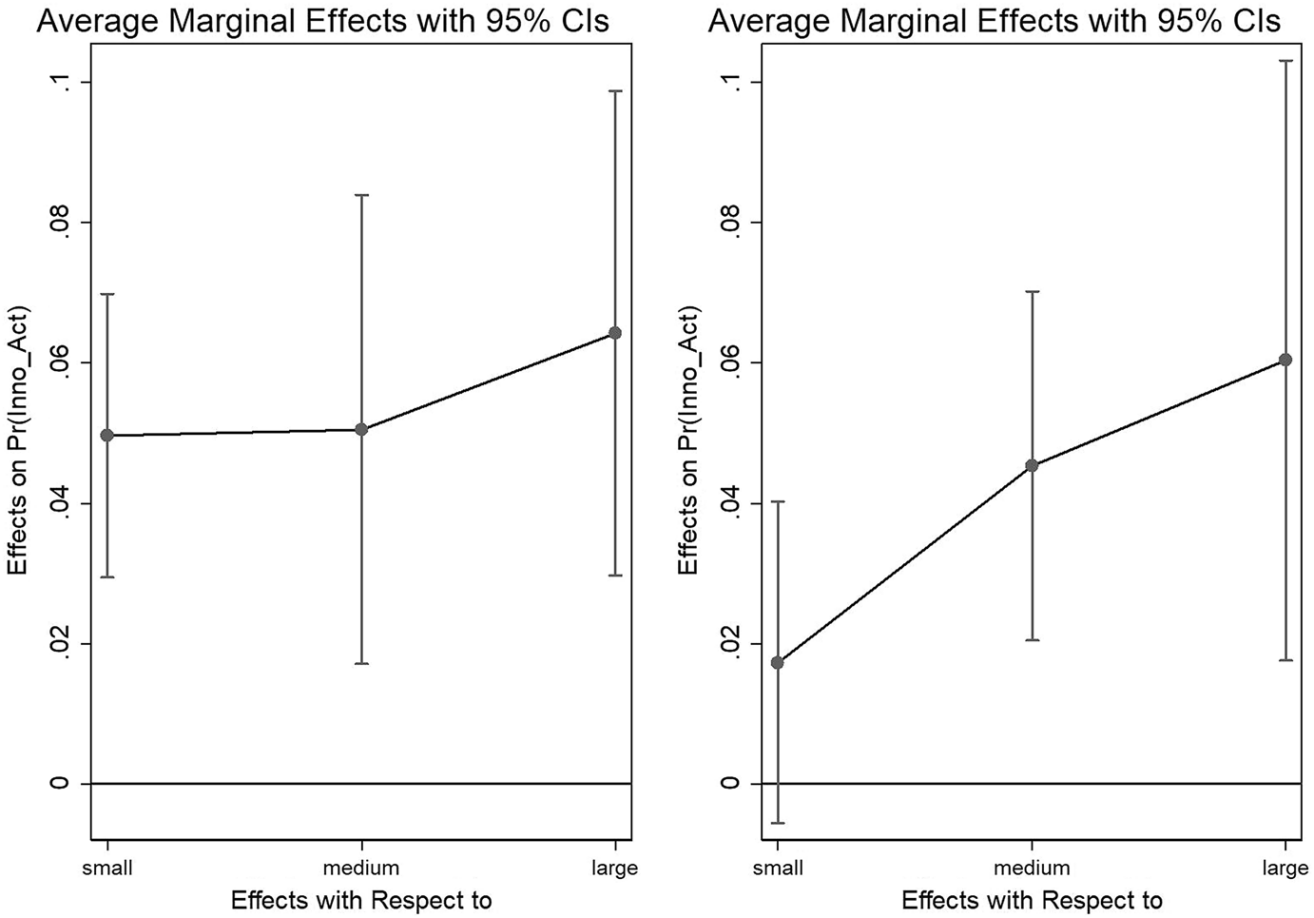
Marginal effects on predicted probability of firm’s innovation activities with respect to firm size before (left) and after (right) the crisis. Note: Marginal effects show if the difference between subgroups of a categorical variable are significant. Here, the reference group is “micro firms”. Source: European Bank for Reconstruction and Development

The estimates suggest a positive and significant relationship between firm size and firm innovation: the odds to innovate increase with size. Large firms have 27% higher odds to innovate compared to micro firms, whereas the odds to innovate decrease around 11 percentage points for small firms. Looking at the marginal effects of firm size on firm’s predicted innovation activities and comparing the two surveys (see Fig. 2,^8^), we can see that before the GFC small firms are more likely to innovate compared to micro firms, but there are no substantial differences to medium and large companies. However, after the GFC large firms have a higher predicted likelihood to innovate. Although it appears that small firms were innovating before the GFC, our overall results suggest a shift of innovation activities from small to large firms, indicating a process towards creative accumulation during and after the crisis. This is plausible as larger firms have more resources and are thus more resistant to a crisis and continue to innovate. Thus, we can confirm our first hypothesis.

**Fig. 3:**
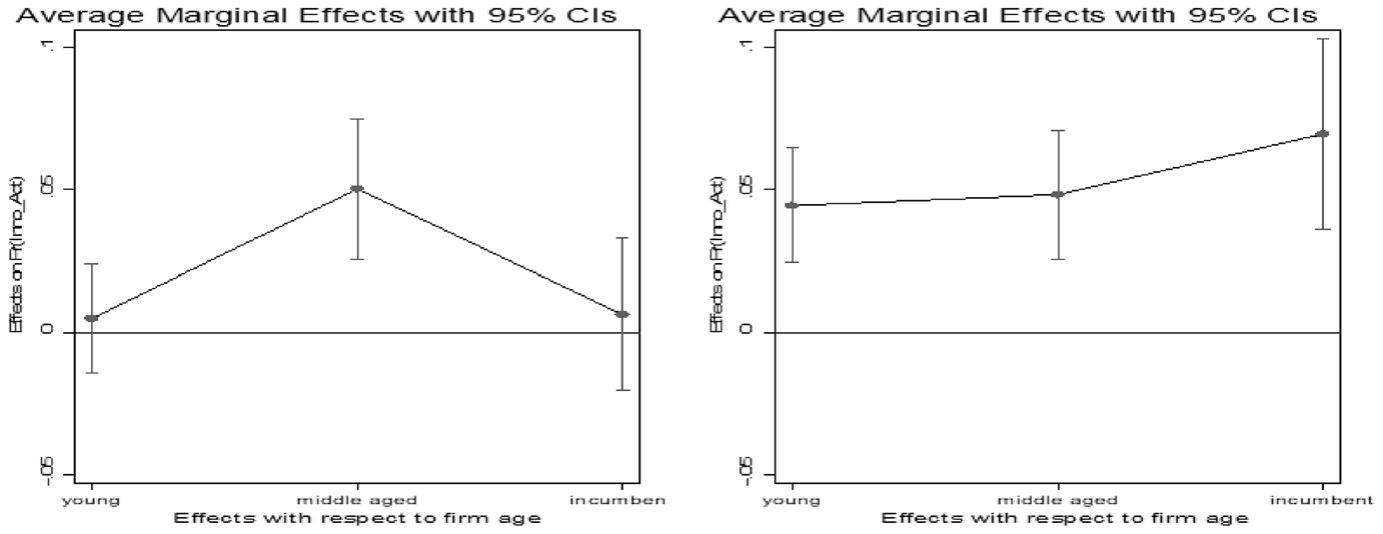
Marginal effects on predicted probability of firm’s innovation activities with respect to firm age before (left) and after (right) the crisis. Note: Marginal effects show if the difference between subgroups of a categorical variable are significant. Here, the reference group is “start-up firms” Source: European Bank for Reconstruction and Development

Turning to firm age, the results similarly suggest a positive and significant relationship between firm age and innovative activities. Again, the odds to innovate increased with age. Incumbent firms have 34% higher odds and middle-aged firms have 29% higher odds to innovate compared to start-up firms, while young firms have 16% higher odds to innovate. Figure 3 shows the marginal effects of firm age on firm’s predicted innovation activities for both surveys. According to Fig. 3, before the GFC middle aged firms are more likely to innovate compared to start-up firms, while after the GFC also young firms and incumbent firms have a higher probability to innovate. These findings indicate that incumbent firms which in general perform innovation activities more routinely, innovate less affected by the crisis. Once again, this confirms our hypothesis. However, we also see a rise in the likelihood to innovate among young firms. This could indicate a behavior of creative destruction. Hence, we cannot fully rule out our second hypothesis.

**Fig. 4:**
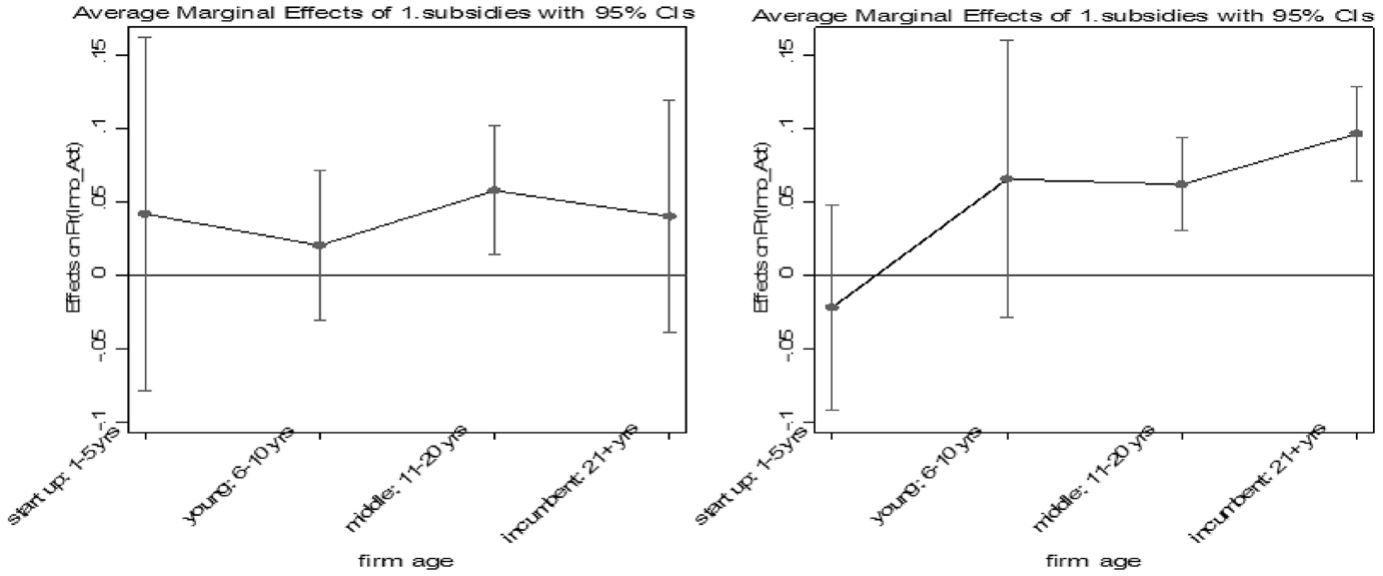
Marginal effects on predicted probability of firm’s innovation activities with respect to receiving subsides across firm size before (left) and after (right) the crisis. Note: Marginal effects show if the difference between subgroups of a categorical variable are significant. Here, the reference group is “firm with no loan” Source: European Bank for Reconstruction and Development

Concerning our third hypothesis, our results indicate that firms with access to finance such as a current loan or receiving subsidies have indeed higher odds to innovate compared to firms that do not have access to these financial resources. Firms that receive governmental subsidies over the last 3 years have 35% higher odds to innovate than those that do not. As in transition economies the institutional environment often fosters the success of incumbent firms (Golikova & Kuznetsov, 2017), we compare in Fig. 4 the probability to innovate of firms that are receiving governmental subsidies across firm age before and after the GFC. Before the GFC, firms have (disregarding age) about the same level of likelihood to innovate. After the crisis, older subsidized companies are more likely to innovate. This result indicates that older companies may receive more government support. A possible reason might be that older firms are receiving more publicly funded support because they have a stronger political network or on the basis of the concept ‘too big to fail’ incumbent firms get more public support.

Firms with a current loan have 29% higher odds than those that do not. Thus, it appears that firms with access to finance are more likely to innovate, which confirms our third hypothesis.

**Fig. 5:**
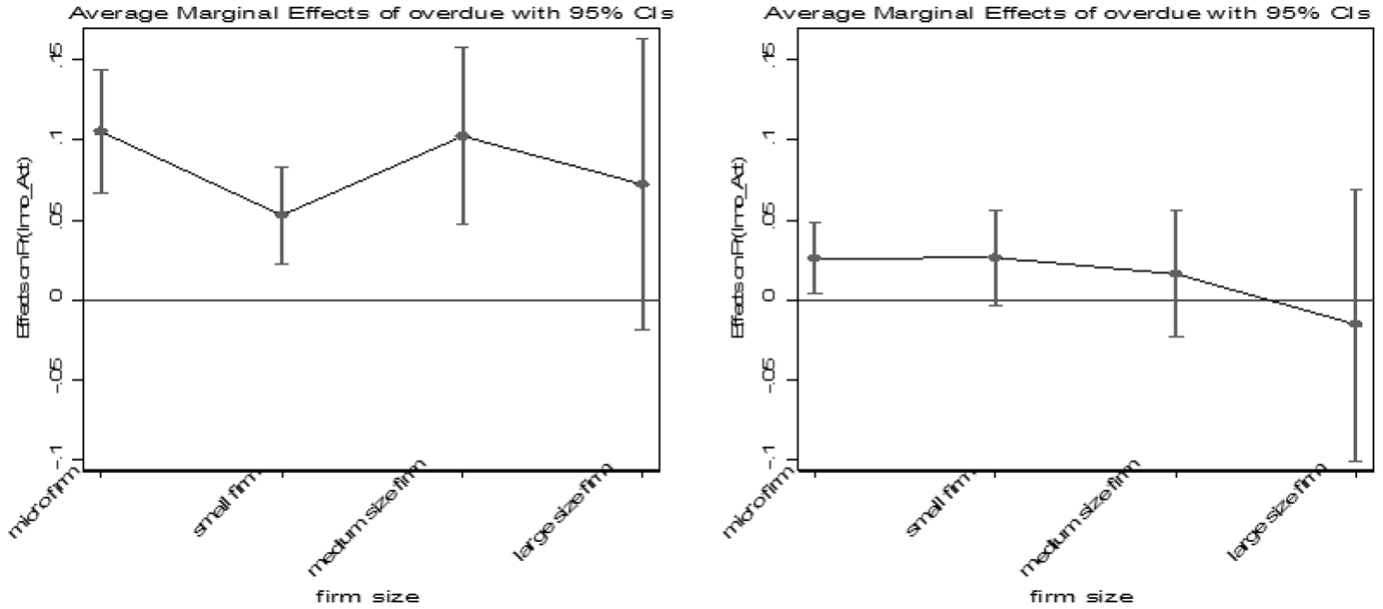
Marginal effects on predicted probability of firm’s innovation activities with respect to overdue payments across firm size before (left) and after (right) the crisis. Note: Marginal effects show if the difference between subgroups of a categorical variable are significant. Here, the reference group is “no overdue payments” Source: European Bank for Reconstruction and Development

Furthermore, firms that are doing well and increase their number of employees have 29% higher odds to innovate compared to those who maintain their employee number.^9^ However, decreasing the number of employees is not significant. Interestingly, those firms with issues to access finance and firms with financial constraint in form of overdue payments have as well higher odds to innovate. How can this be? Companies that state accessing finance is a great obstacle have 26% higher odds to innovate than those with moderate difficulties. In addition, firms with overdue payments have 25% higher odds compared to firms that do not. What seems counterintuitive at first sight, becomes clearer on closer examination. Comparing the marginal effects of having overdue payments across firm size (see Fig. 5), it becomes visible that after the crisis the likelihood of firms (disregarding size) with overdue payments to innovate decreases. These results suggest that innovating firms are more likely to face financial constraints than firms that do not pursue innovation activities. These findings are consistent with Mateut (2018) and Gorodnichenko and Schnitzer (2013).

**Fig. 6:**
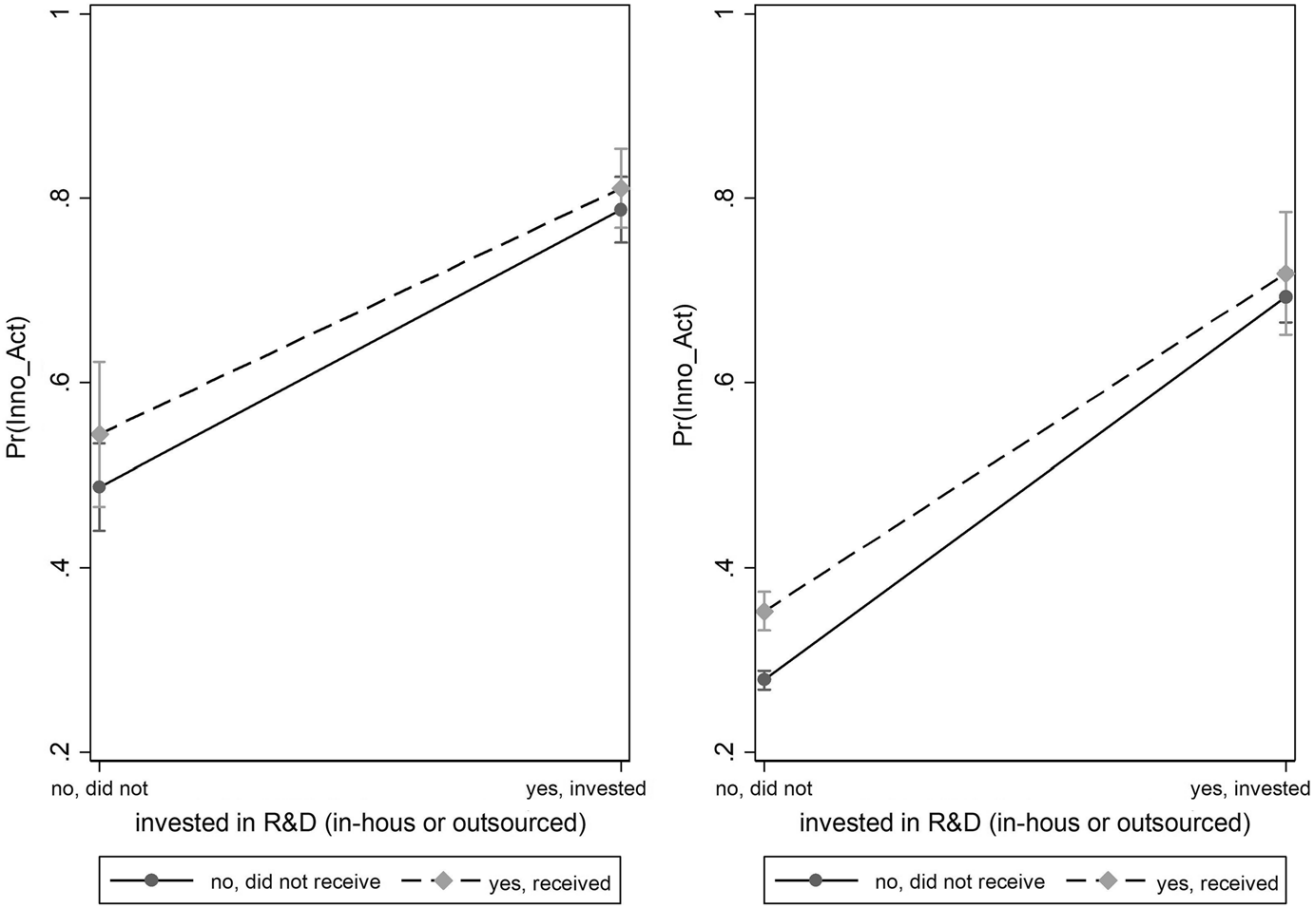
Marginal effects on predicted probability of firm’s innovation activities with respect to receiving subsidies and spending on R&D before (left) and after (right) the crisis. Note: Marginal effects show if the difference between subgroups of a categorical variable are significant. Source: European Bank for Reconstruction and Development

With respect to hypothesis 3a, we find that access to finance in the form of subsidies in combination with R&D leads to a higher predicted innovation probability before and after the financial crisis (see Fig. 6). However, the innovation probability of firms that do not receive subsidies drops by half after the crisis. A similar picture emerges when looking at access to credit and R&D. Due to the high degree of similarity, only one figure is presented here. It seems that access to financial sources supports companies' innovation activities. Although firms that invest in R&D without financial support from subsidies also have a higher predicted probability of innovating.

**Fig. 7:**
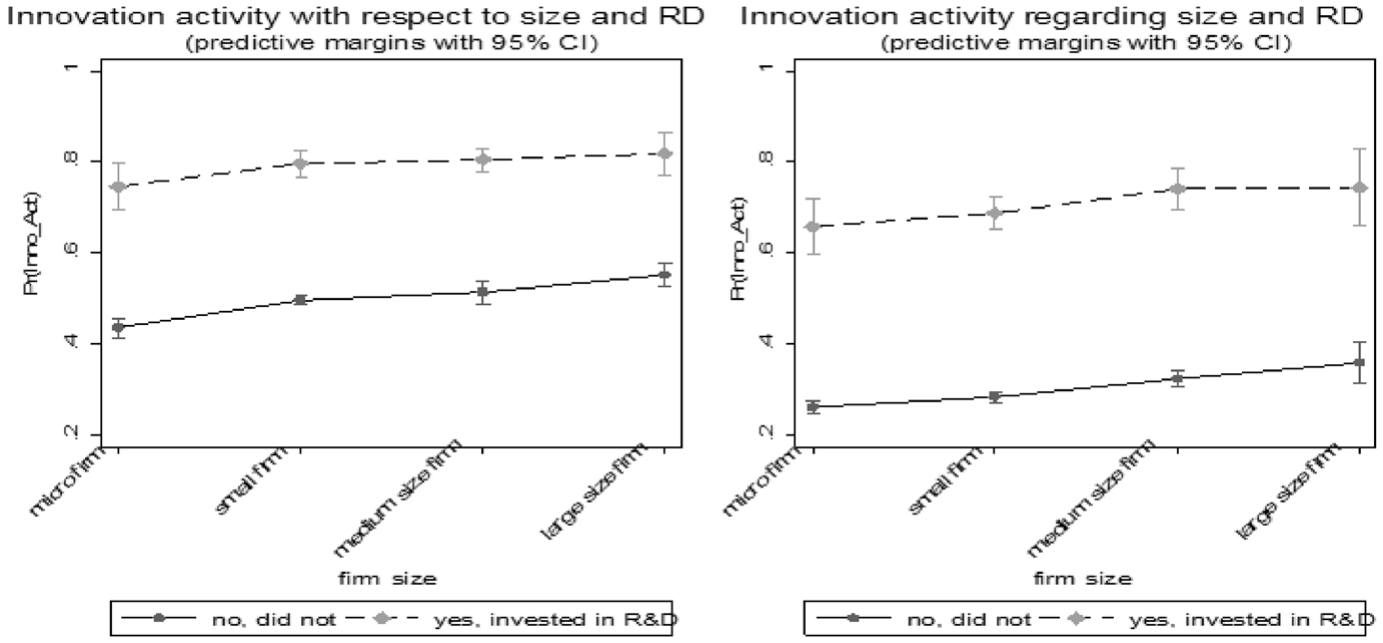
Predicted probability of firms’ innovation activity depending on R&D activities across firm size before (left) and after (right) the crisis. Source: European Bank for Reconstruction and Development

Moving to our control variables, our findings show that R&D activities are an important input-factor for innovation. This is in line with the results of Gogokhia and Berulava (2021). The odds to innovate are over five times higher for companies involving themselves in R&D than those that do not. These results are in line with Archibugi et al., (2013a, b). Comparing firms’ R&D activities across firm size before and after the GFC shows that R&D stabilizes innovation across firm sizes (see Fig. 7). We see that (disregarding the firm size), firms which didn’t invest into R&D have a lower level of probability to engage in innovation activity. Whereas the probability of R&D investors only decreases by 5 percentage points. A similar picture appears comparing firms’ R&D activities across firm age before and after the GFC.^10^ These results suggest that companies that invest in R&D innovate more continuously throughout a crisis.

Regarding internationalization, our findings show that foreign firms have 30% higher odds to innovate compared to domestically owned ones. Furthermore, companies involved in the export business have almost 40% higher odds to innovate than those that are not. These findings are in line with Paunov (2012) and show that internationalization helps to stimulate innovation in transition economies. Human capital measured as the share of employees with a university degree makes innovation activities within firms more likely. Firms where a majority of employees hold a university degree have 53% higher odds to innovate than firms with no university-trained employees. These results support the premise that innovation knowledge is impersonated in skilled workers and should not be dismissed due to a crisis (Hall & Lerner, 2010; Paunov, 2012). Besides, we control the firm’s background whether the firm was privatized or run privately from the start. Our results suggest that privatized firms have around 20% fewer odds to engage in innovative activities compared to firms created by a joint venture etc., while the difference between joint ventures and private firms since their start is not significant. This could imply that privatized firms maybe offer a less creative environment and, thus, have less odds to innovate.

### Robustness checks

Although firm age is a good measure for a firm’s experience and knowledge base, it does not necessarily mean that the firm’s manager is as experienced as firm age implies. Furthermore, Amore (2015) demonstrated that past experience shapes firms’ innovation decisions during crises. Therefore, we additionally use an alternative measure of manager experience.

We present the robustness checks in column 2 of Table 2. Overall, our findings still hold. The odds to innovate increase by 3 percentage points for medium sized firms and by 5 percentage points for large firms compared to the baseline estimations. Firms run by managers with 11–20 years of experience have 32% higher odds compared to firms with unexperienced managers. Though, the odds to innovate decrease by 5 percentage points for firms that employ managers with over 20 years of experience. This indicates that with higher age managers are getting less eager to innovate. Nevertheless, it supports our findings above, during and after the crisis innovation activities across firms shifted and became more concentrated in experienced firms.

So far, we have focused on product or process innovation as well as on pooled wave. Table 3 shows the results in column (1) of product/process innovations, as the 2009 BEEPS survey does not allow a delimitation of process innovations. Column (2) analyses the 2012 wave separately with reference to product/service innovation or, in the case of column (3), to process innovation. Overall, our findings remain similar. Slight differences appear when analyzing Beeps 2009 individually. Differences within the categories firm age are less significant. However, this confirms the results of the marginal plots presented above. Before the crisis, small firms as well as middle aged firms were more likely to innovate. However, after the crisis a shift of innovation activities happens towards large and incumbent firms having the highest odds to innovate which can be seen in the results of column (2). This is indicating a process towards creative accumulation. When only focusing on process innovation, we can see that the odds to innovate increase even more with age and size. This makes sense as process innovation conducted to reduce costs, to increase output or quality is more common among larger firms. This is in line with the results of Paunov (2012).

**Table 3:** Logit estimation results of BEEPS waves separately by innovation type

	(1)	(2)	(3)
	Product/process 2009	Product 2012	Process 2012
Size: small firm	1.280***	1.027	1.231**
	(0.0650)	(0.0623)	(0.0858)
Size: medium firm	1.286**	1.205**	1.417***
	(0.109)	(0.0844)	(0.110)
Size: large firm	1.378***	1.297*	1.690***
	(0.122)	(0.178)	(0.194)
Age: young firm (6–10 years)	0.997	1.311***	1.383***
	(0.0492)	(0.0897)	(0.132)
Age: middle aged (11–20 years)	1.257***	1.317***	1.428***
	(0.0747)	(0.0713)	(0.136)
Age: incumbent (> 20 years)	1.035	1.545***	1.534***
	(0.0724)	(0.132)	(0.196)
RD activities	4.557***	5.128***	5.493***
	(0.266)	(0.296)	(0.350)
Subsidies	1.222**	1.378***	1.384***
	(0.117)	(0.0751)	(0.125)
Current loan	1.248***	1.288***	1.351***
	(0.0583)	(0.0910)	(0.0688)
Overdue	1.437***	1.170**	1.215***
	(0.0763)	(0.0758)	(0.0691)
Access finance: no/minor obstacle	1.007	0.924	1.082
	(0.0806)	(0.0618)	(0.0758)
Access finance: great obstacle	1.239***	1.161**	1.442***
	(0.0752)	(0.0735)	(0.103)
N	9015	12,418	12,497
Pseudo R^2^	0.153	0.159	0.172
Log likelihood	− 5235.0208	− 5987.5693	− 5304.9642
LR Chi^2^	1891.62	2262.15	2200.39
Prob > Chi^2^	0.000	0.000	0.000

Reference groups are as follows: for firm size: micro firms; age: 1–5 years; employee growth: unchanged; access finance: moderate obstacle. All controls included like in Table 2. Time controls, sector, and country fixed-effects included. Exponentiated coefficients: to better interpret our results, we transform the coefficients into odds ratio; standard errors in parentheses are clustered at the sector × wave level, *p < 0.10, **p < 0.05, ***p < 0.001. Source: European Bank for Reconstruction and Development

To account for the complementary nature of product and process innovations (Mantovani, 2006), we also apply a bivariate probit estimation. Table 7 presents the marginal effects at the means. Overall, our results are comparable. For both, product and process innovation, the probability that a firm will innovate increases with size and age. The estimated effect of large firm is higher for process innovation with 27% compared to 15% for product innovation. R&D activities positively affect the probability that firms innovate either in products or processes. Further, firms with access to finance in form of subsidies and a loan have a higher probability to innovate. The Wald test shows a significant correlation between the error terms, but the estimation yields very similar results. The magnitude of the marginal effects was compared with a probit estimate (see Table 8). Here, too, there are no overly large differences.

### Further analysis: subsamples

To account for the different levels of development of the countries considered here, we use the possibility that BEEPS allows for the comparison of cross-country variations. Thus, we estimate two subsamples with respect to EU-membership (see Table 4, Colum 1 and 2). Among EU countries, young firms have with 33% the highest odds to innovate compared to start-up firms. While in non-EU countries middle-aged firms have the highest odds to innovate. Once again, the difference in odds regarding firm size is noticeable. In transition countries without an EU membership, odds increase much more with firm size compared to EU transition countries. This could be related to the institutional environment in these countries which often fosters the success of larger firms. Moreover, we find that being involved in R&D activities increases the likelihood in both country groups. Although the relation is stronger in non-EU countries. In both country groups, having a current loan increases the likelihood to innovate. However, the odds to innovate are 20 percentage points higher among non-EU members. Hence, it appears that in these countries access to finance has a higher importance to innovating firms.

**Table 4:** Logit estimations of pooled subsamples with respect to EU-membership and sectors

	(1)	(2)	(3)	(4)
	EU transition countries	None-EU countries	Manufacturing	Service
Size: small firm	1.066	1.195***	1.076	1.197***
	(0.0643)	(0.0544)	(0.0863)	(0.0516)
Size: medium firm	1.104*	1.288***	1.099	1.315***
	(0.0983)	(0.0833)	(0.102)	(0.0855)
Size: large firm	1.072	1.361**	1.300*	1.178**
	(0.123)	(0.133)	(0.196)	(0.0830)
Age: young firm (6–10 years)	1.329**	1.131**	1.183**	1.134**
	(0.141)	(0.0609)	(0.0882)	(0.0703)
Age: middle aged (11–20 years)	1.218**	1.340***	1.388***	1.219***
	(0.113)	(0.0614)	(0.105)	(0.0454)
Age: incumbent (> 20 years)	1.292**	1.290***	1.360**	1.314**
	(0.166)	(0.0852)	(0.134)	(0.116)
RD activities	4.588***	5.704***	5.924***	4.774***
	(0.343)	(0.486)	(0.454)	(0.433)
Subsidies	1.436***	1.298**	1.321**	1.393***
	(0.115)	(0.132)	(0.116)	(0.0931)
Current loan	1.118*	1.386***	1.353***	1.245***
	(0.0674)	(0.0578)	(0.0650)	(0.0590)
Overdue	1.290**	1.247***	1.275***	1.246***
	(0.103)	(0.0568)	(0.0821)	(0.0571)
Access finance: no/minor obstacle	1.029	0.974	1.089	0.911*
	(0.0937)	(0.0450)	(0.0695)	(0.0444)
Access finance: great obstacle	1.203*	1.250***	1.413***	1.149**
	(0.121)	(0.0648)	(0.0838)	(0.0654)
N	5969	15,496	8665	13,120
Pseudo R^2^	0.148	0.193	0.172	0.172
Log likelihood	− 3517.3949	− 8450.2012	− 4790.0005	− 7174.0415
LR Chi^2^	1224.61	4042.48	1985.20	2973.09
Prob > Chi^2^	0.000	0.000	0.000	0.000

The dependent variable is binary standing for process or product/service innovation activities. Reference groups are as follows: for firm size: micro firms; age: 1–5 years; employee growth: unchanged; access finance: moderate obstacle. All controls included like in Table 2. Time controls, sector, and country fixed-effects included. Exponentiated coefficients: to better interpret our results, we transform the coefficients into odds ratio; standard errors in parentheses are clustered at the sector × wave level, *p < 0.10, **p < 0.05, ***p < 0.001. Source: European Bank for Reconstruction and Development

To see if industry specialization plays a role, we further divide the sample into manufacturing and service sector. Since the nature of innovation in the service sector can be different from manufacturing (Pellegrino & Piva, 2020). Column 3 and 4 of Table 4 provide the estimation results. The results remain similar. In both sectors, older and larger firms are more likely to innovate compared to smaller and younger companies. Firms with access to finance in form of subsidies and loans have as well a higher likelihood to innovate. This applies for manufacturing and service firms.

## Conclusion

This study has investigated the innovation behavior of companies in 29 transition economies within CEE and the CIS and compares their innovation activities before and after the GFC 2008/2009. Using BEEPS data, we investigated over 25,000 firms in two pooled surveys conducted in the years 2009 and 2012. Overall, we find strong empirical support for a shift of innovation activities from small to incumbent and large companies, indicating the Schumpeterian phenomenon of creative accumulation after the crisis. However, young firms also have a higher likelihood to innovate after the crisis, whereas we cannot say the same regarding small firms. Regarding financial measures, we find that firms that have access to finances in form of a loan or subsidies are more likely to innovate. Furthermore, our findings highlight the importance of R&D activities within companies as these have a significant stabilization effect on firms’ innovation behavior in times of crisis.

The studied countries have gone through a radical transition process from a planned to a market economy and have reached different degrees of modernization and technological capability. The market-based innovation systems, even in EU transition economies, are relatively young and still developing a technological profile, networks between actors, and institutions. It is plausible to assume that an external shock hits these countries’ innovation activity quite hard. In the light of these considerations, it is insightful to observe that a major Schumpeterian theoretical prediction, creative accumulation, holds true. Creative destruction is not fully confirmed, which is probably an indication for the still weak or emerging start-up milieus in transition economies. Policy makers should be encouraged by our findings to support research and development activities in firms, which is a basis for innovative activities and helps firms to weather the crisis.

Our findings mostly align with what is found in the empirical literature. Creative destruction and creative accumulation are two co-existing scenarios and a clear distinction between those two is not possible. This is also reflected in the findings of the empirical literature. While some studies show a stronger tendency to creative destruction during the GFC 2008/2009 in Europe (Archibugi et al., 2013a, b), most findings suggest that established companies are more likely to innovate during this economic downturn which points to creative accumulation (Correa & Iootty, 2010; Paunov, 2012; Teplykh, 2018). In this respect, our paper supports these findings.

As every empirical analysis, our investigation is not without limitations. Firstly, companies that did not survive the crisis are not in the data set. However, we are mainly interested in the innovation behavior of companies that survived the crisis or were created during the crisis. Thus, this limitation does not undermine our results; it is just that we cannot say anything about the firms that dropped out of the market. Hence, we cannot answer the question whether non-surviving firms left the market because they were less innovative and thus less successful or they might have exited because innovation activities depleted their financial resources. Secondly, due to data restrictions, we cannot control for the differences of maintaining, increasing or decreasing innovation activities only for the type of firms that do innovate in times of crisis. Therefore, we are only able to observe the aggregated shifts in firms’ innovation behavior. This limitation stresses the need of further research on this matter. Given the overall decline in innovation activities during the GFC, the question remains whether this decline in innovation and R&D is less pronounced for larger firms or whether larger firms are using innovation as a coping strategy to get through the crisis. Finally, even though self-reported measurements provide in our case earlier mentioned advantages, we are aware that self-reported data are more vulnerable to measurement error and cultural bias.

## Data Availability

The data used in this paper are publicly available at the website of European Bank for Reconstruction and Development.

## Appendix

See Tables 5, 6, 7 and 8.

**Table 5:** Correlation matrix (pooled sample)

	Firm size	Firm age	Manager experience	R&D activities	Current loan	Subsidies	Employee growth	Overdue	Access finance	Foreign owned	Background	Human capital	Export
Firm size	1												
Firm age	0.244	1											
Manager experience	0.078	0.419	1										
R&D activities	0.318	0.112	0.074	1									
Current loan	0.304	0.123	0.066	0.275	1								
Subsidies	0.251	0.173	0.077	0.288	0.336	1							
Employee growth	− 0.067	0.301	0.132	− 0.102	− 0.082	− 0.018	1						
Overdue	0.158	0.074	0.029	0.193	0.339	0.187	− 0.050	1					
Access finance	0.035	0.007	0.003	0.119	0.196	− 0.012	0.048	0.122	1				
Foreign owned	0.337	− 0.048	− 0.065	0.176	0.026	0.086	− 0.051	0.082	− 0.109	1			
Background	0.417	0.348	0.090	0.108	0.023	0.154	0.242	− 0.040	0.038	0.223	1		
Human capital	− 0.008	− 0.139	− 0.127	0.019	− 0.142	− 0.108	− 0.072	− 0.037	0.023	0.040	− 0.056	1	
Export	0.368	0.186	0.104	0.397	0.313	0.379	− 0.006	0.203	0.017	0.400	0.165	− 0.100	1

Source: European Bank for Reconstruction and Development

**Table 6:** Overview of firms’ number (and share) by industry in pooled sample

Variables	Industry Label	Freq	Percent	
sec15-16	Food and tobacco	1971	7.82	
sec17-19	Textiles, clothing, leather	1399	5.55	
sec20-22	Wood, paper, printing	1198	4.75	
sec23-26	Coke, chemicals, rubber, plastic	1787	7.09	
sec27-28	Metals	1122	4.45	
sec29_34-35	Machinery	1061	4.21	
sec30-33	Electronics, instruments	596	2.36	
sec36-37	Other manufacturing	531	2.11	
sec45	Construction	2380	9.44	
sec50-52	Retail, wholesale	10,112	40.11	
sec55	Transport	1134	4.50	
sec60-64	Hotel, restaurant	1379	5.47	
sec72	IT	419	1.66	
Total		25,089	100	

Source: European Bank for Reconstruction and Development

**Table 7:** Bivariate probit estimation results

	(1a) Product innovation		(1b) Process innovation
Small firm	0.0120		0.113**
	(0.0325)		(0.0345)
Medium firm	0.105**		0.179***
	(0.0447)		(0.0473)
Large firm	0.151**		0.277***
	(0.0754)		(0.0784)
Age: young firm (6–10 years)	0.152**		0.229***
	(0.0487)		(0.0579)
Age: middle aged (11–20 years)	0.154**		0.113**
	(0.0475)		(0.0345)
Age: incumbent (> 20 years)	0.242***		0.179***
	(0.0550)		(0.0473)
RD activities	0.984***		1.003***
	(0.0410)		(0.0405)
Subsidies	0.182***		0.166***
	(0.0487)		(0.0497)
Current loan	0.152***		0.170***
	(0.0298)		(0.0313)
Overdue	0.0899**		0.100**
	(0.0299)		(0.0314)
Access finance: no/minor obstacle	-0.0473		0.0464
	(0.0346)		(0.0372)
Access finance: great obstacle	0.0907**		0.211***
	(0.0416)		(0.0437)
N	12,396		12,396
Wald Chi^2^	2979.56		2979.56
Prob > Chi^2^	0.0000		0.0000
Wald test of rho = 0		Chi^2^(1) = 1200.93 Prob > Chi^2^ = 0.0000	

Marginal effects at the means are reported. Reference groups: for firm size: micro firms; age: 1–5 years; access finance: moderate obstacle. All controls included like in Table 2. Country and industry fixed-effects are included. Standard errors in parentheses are clustered at sector × wave level. Robust standard errors in parentheses **p* < 0.10, ***p* < 0.05, ****p* < 0.001. Source: European Bank for Reconstruction and Development

**Table 8:** Probit estimation results

	(1) Product innovation	(2) Process innovation
Small firm	0.0142	0.113**
	(0.0325)	(0.0348)
Medium firm	0.106**	0.180***
	(0.0448)	(0.0475)
Large firm	0.149**	0.275***
	(0.0752)	(0.0785)
Age: young firm (6–10 years)	0.153**	0.181***
	(0.0487)	(0.0515)
Age: middle aged (11–20 years)	0.155**	0.199***
	(0.0475)	(0.0504)
Age: incumbent (> 20 years)	0.247***	0.239***
	(0.0550)	(0.0586)
RD activities	0.989***	1.006***
	(0.0416)	(0.0412)
Subsidies	0.186***	0.173***
	(0.0487)	(0.0501)
Current loan	0.149***	0.169***
	(0.0299)	(0.0314)
Overdue	0.0882**	0.105***
	(0.0299)	(0.0317)
Access finance: no/minor obstacle	− 0.0464	0.0406
	(0.0348)	(0.0374)
Access finance: great obstacle	0.0862**	0.202***
	(0.0418)	(0.0440)
N	12,418	12,420
Pseudo R^2^	0.159	0.172

Marginal effects at the means are reported. Reference groups: for firm size: micro firms; age: 1–5 years; access finance: moderate obstacle. All controls included like in Table 2. Country and industry fixed-effects are included. Standard errors in parentheses are clustered at sector × wave level. Robust standard errors in parentheses **p* < 0.10, ***p* < 0.05, ****p* < 0.001. Source: European Bank for Reconstruction and Development

## Footnotes

1 Both surveys contain 17 countries from CEE: Albania, Bosnia and Herzegovina, Kosovo, FYR Macedonia, Montenegro, Serbia, and the EU member states Bulgaria, Croatia, Czech Republic, Estonia, Hungary, Latvia, Lithuania, Poland, Romania, Slovak Republic and Slovenia, another 11 countries from the former Soviet Union: Armenia, Azerbaijan, Belarus, Georgia, Kazakhstan, Kyrgyzstan, Moldova, Russia, Tajikistan, Ukraine and Uzbekistan, as well as Mongolia and Turkey.

2 The countries Cyprus and Greece were only surveyed in BEEPS 2012.

3 The manufacturing sector includes manufacturing and agro-processing, but not primary industries such as mining or agriculture. The service sector includes retail, wholesale, IT and repair services, hotel and restaurants as well as transportation and communication services.

4 This three-year period covers 2005–2007 in BEEPS 2009 and 2009–2011 in BEEPS 2012, but it should be noted that Russian companies were surveyed one year earlier in the 2012 survey. Thus, in this case, the innovation period covers 2008–2010.

5 According to OECD (2017) firm sizes can be subdivided into micro (fewer than 10 employees), small (10–49 employees), and medium-sized enterprises (50 to 249 employees). Large enterprises employ 250 or more people.

6 Archibugi et al., (2013a, b) are using R&D expenditures in their analysis. Due to a large amount of missing data for R&D expenditures, we decided against using this indicator. We are aware that the binary R&D variable has its limitations.

7 Since the Beeps 2009 definition of product innovation explicitly refers to changes in the process, we combined it with the queries of process or product innovation in Beeps 2012 on the independent variable (product or process innovation).

8 The corresponding regression tables of the marginal effects are available upon request.

9 As an alternative to employee growth, we used sales growth in our analysis. The findings are similar. Companies that report an increase in turnover are more likely to be innovative. Due to the high numbers of missing that occurred when creating this measure, we decided not to include sales growth in our main analysis.

10 Due to the strong similarity, we have only included one figure. The other illustration is available upon request.
